# Thermal Degradation and Flame Resistance Mechanism of Phosphorous-Based Flame Retardant of ABS Composites Used in 3D Printing Technology

**DOI:** 10.3390/ma18133202

**Published:** 2025-07-07

**Authors:** Rafał Oliwa, Katarzyna Bulanda, Mariusz Oleksy

**Affiliations:** Faculty of Chemistry, Department of Polymer Composites, Rzeszow University of Technology, Al. Powstańców Warszawy 6, 35-959 Rzeszów, Poland; oliwa@prz.edu.pl (R.O.); molek@prz.edu.pl (M.O.)

**Keywords:** FDM, ABS, flame resistance, aryl phosphate flame retardant, thermal stability

## Abstract

As part of the work, polymer composites dedicated to rapid prototyping were developed, especially for 3D printing using the material extrusion technique. For this purpose, a polymer matrix was selected, which was an acrylonitrile-butadiene-styrene (ABS) terpolymer and a flame retardant, which was tetrakis (2,6-dimethylphenyl)-m-phenylenebisphosphate, commercially known as PX200. The effect of the presence and amount (5, 10, 15 and 20 wt.%) of the introduced additive on the rheological properties, structural properties, flammability (limiting oxygen index, LOI; UL94) and flame retardant properties (microcone calorimeter, MLC) of ABS-based composites was investigated. In addition, the mechanism of thermal degradation and flame resistance was investigated using thermogravimetric analysis, TGA and Fourier transform infrared spectroscopy, FT-IR of the residue after the MLC test. In the first part of the work, using the author’s technological line, filaments were obtained from unfilled ABS and its composites. Samples for testing were obtained by 3D printing in Fused Deposition Modeling (FDM) technology. In order to determine the quantitative and qualitative spread of fire and the effectiveness of the phosphorus flame retardant PX200 in the produced composites, the Maximum Average Rate of Heat Emission (MARHE); Fire Growth Rate Index (FIGRA); Fire Potential Index (FPI) and Flame Retardancy Index (FRI) were determined. Based on the obtained results, it was found that the aryl biphosphate used in this work exhibits activity in the gas phase, which was confirmed by quantitative assessment using data from a microcone calorimeter and non-residues after combustion and thermolysis at 700 °C. As a result, the flammability class did not change (HB40), and the LOI slightly increased to 20% for the composite with 20% flame retardant content. Moreover, this composite was characterized by the following flammability indices: pHRR = 482.9 kW/m^2^ (−40.3%), MARHE = 234 kW/m^2^ (−40.7%), FIGRA = 3.1 kW/m^2^·s (−56.3%), FPI = 0.061 m^2^·s/kW (+64.9%), FRI = 2.068 (+106.8%).

## 1. Introduction

3D printing, also known as spatial printing or additive technology, is a method of producing three-dimensional elements by gradually applying material layer by layer. The process uses Computer Aided Design (CAD). A 3D model of the manufactured element is created in an appropriate program. The model, saved as a file, is sent to a 3D printer in which it is divided into layers and then the printing phase takes place by applying layers of material one on top of the other. Depending on the selected method, material and type of printer, this process can be done in different ways [1,2]. According to the ISO/ASTM 52900 Standard (2021) [3], there are seven basic categories of additive technologies: Binder Jetting, Directed Energy Deposition, Material Jetting, Material Extrusion, Powder Bed Fusion, Sheet Lamination, Vat Polymerization.

**Figure 1:**
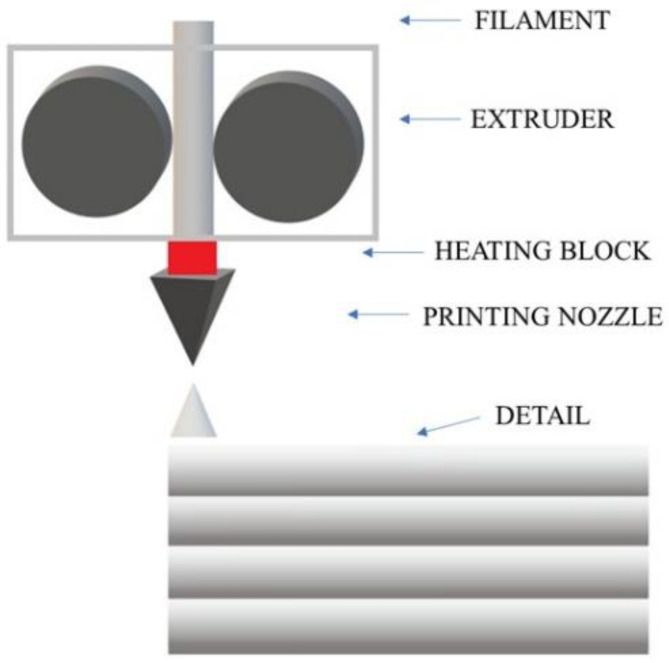
Diagram of the construction of a 3D printer using the FDM method.

The most common technique conquering the 3D printing market is FDM, Fused Deposition Modeling. This method is widely used due to its many advantages, such as low material and production costs, ease of use, low maintenance costs, the ability to operate at low temperatures, and the ergonomics of a 3D printer both at home and in a laboratory or workshop [4]. In FDM technology, a thermoplastic polymer material in the form of a thin filament is heated in the extruder head above its melting temperature (Figure 1). Then, through a nozzle, the melted material is extruded onto the heated printer’s working platform. The extruder head and nozzle move horizontally along a path selected by the user until the first layer of the element is applied. The platform is lowered by a height corresponding to the thickness of the selected layer and another layer is applied on top of the first layer. The process continues until all layers of the selected model are applied [5]. Before the next layer is applied, the previous one must cool down sufficiently and thus solidify to provide a sufficiently solid base for the rest of the print. However, it should not be completely solidified so that the next layer bonds well with the previous one. The adhesion of the applied layer is increased by the aforementioned heating of the working platform to a specific temperature depending on the selected material. In this way, the hardening process of the layers is optimized, which occurs immediately after the material is extruded from the nozzle [5,6,7,8,9,10].

Each filament production is characterized by different requirements for their production. Filament diameters are standardized—the three most commonly used are 1.75 mm, 2.85 mm and 3 mm. The diameter and quality of the filament are influenced by extrusion parameters, such as extrusion temperature, extrusion speed, nozzle diameter and cooling conditions. Extrusion parameters are crucial for forming a filament with a constant cylindrical cross-section and dimensional precision during production [11]. A standard single- or twin-screw extruder with a nozzle of the appropriate diameter is used for filament production, through which previously prepared plastic granulate is extruded. The screw rotation speed, pressure and extrusion temperature are key parameters during extrusion and must be controlled to obtain a filament with the required diameter. If the appropriate parameters are not met, the extruded filament may break or buckling may occur when the extrusion pressure is lower or higher than the critical load that the fiber can withstand [12].

When selecting the right polymer to produce a filament, its rheological, mechanical, structural, physical and chemical properties should be taken into account. For example, viscosity—one of the basic properties of polymers and is important during 3D printing, as it affects the maintenance of the desired flow properties of the material during its transport from the heating chamber through the nozzle to the build platform. The strength of intermolecular bonds between individual polymer chains depends on viscosity. Higher viscosity leads to stronger bonds [13].

The most commonly used materials for the production of filament in FDM technology are polymers: acrylonitrile-butadiene-styrene (ABS) and polylactide (PLA) with a volume strength between 30–100 MPa and a modulus of elasticity in the range of 1.3–3.6 GPa. Other polymer materials used in FDM technology are polycarbonate, polyamide, high-impact polystyrene, polyoxymethylene and others [12,13,14].

Although parts made of polymers using the FDM method have acceptable mechanical strength, they generally have poor fire resistance. Their flammability negatively affects the overall properties of these 3D printed products. Fire is a major threat to most polymers because they are susceptible to combustion due to their hydrocarbon chains. A well-designed plastic product must also be resistant in the event of an unwanted fire [15].

Scientists have been trying to increase the fire resistance of many polymers for decades. Their research has yielded several important conclusions and discoveries. Halogenated flame retardants have been widely used in the past to increase the flame resistance of various polymeric materials. However, their use is currently prohibited due to their undesirable effects, such as environmental pollution and toxicity (by releasing toxic gases). Therefore, it was necessary to develop non-halogenated flame retardants to increase the fire safety of polymeric materials. In this regard, a number of solutions have been designed, such as those based on phosphorus, silicon, metal hydroxides, nitrogen or carbon (fullerenes, carbon nanotubes and graphene) [15].

Among flame retardants, phosphorus-based flame retardants are often used to achieve excellent fire properties of polymers [15,16]. They are effective and environmentally friendly. Phosphorus-based flame retardants can be divided into two classes: organic and inorganic phosphorus flame retardants, which include various compounds in which phosphorus occurs in different oxidation states from 0 to 5. Phosphorus acts in both the condensed and gaseous phases. Due to this dual role, the flame retardant action of phosphorus cannot be described by a single mechanism [17]. The range of organic phosphorus-based flame retardants is very wide and includes products in which phosphorus occurs in different oxidation states, i.e., phosphates, phosphonates, phosphinates and phosphine oxides. The condensed phase action, including the formation of an insulating carbon layer, is the most common mode of action of these flame retardants. The formation of a char layer is the result of the oxidation of phosphorus compounds as well as their interaction with the polymer. Achieving adequate flame retardancy depends on the time between the decomposition of the polymer chains and the action of the flame retardant. The chemical composition of the phosphorus compounds and the thermal and fire reaction of the polymer to the flame together determine the degree of flame retardancy. Therefore, non-halogenated phosphorus-based flame retardants are more dependent on the type of polymer than halogenated flame retardants. In some cases, although few, organophosphates exhibit gas-phase action. These cases are very limited because the evaporation of phosphorus must take place at relatively low temperature and not during the polymer melting process to prevent loss of phosphorus and formation of phosphine [17].

To meet the requirements of flame retardancy, a high level of flame retardant filling of the polymer is required, which entails higher costs, technological difficulties and a decrease in the mechanical properties of the polymer [18].

Flame retardants are often classified based on their mechanism of action. They can limit the spread of flame by physical action (fuel dilution, endothermic cooling, nanoparticle physical barrier effect) or chemical action (flame inhibition, charring, swelling, melt dripping). Some flame retardants, such as metal hydroxides, exhibit almost exclusively physical action in inhibiting the combustion process. However, there is no flame retardant that can act exclusively through chemical mechanisms. They are often combined with several physical mechanisms that delay the fire or stop the combustion process [19]. The combustion process can occur in the condensed or gaseous phase. In the condensed phase, the following of the above-mentioned mechanisms are possible: charring, endothermic cooling effect, melt dripping, swelling, nanoparticle physical barrier effect [16,18]. In the gaseous phase, there are two types of flame retardant action. First, it is the release of inert gases inside the flame during combustion, which dilutes the flammable gases—the so-called fuel dilution. The second mechanism is the generation of radicals resulting from the decomposition of the flame retardant [17]. The previously mentioned phosphorus-based flame retardants operate, among others, on the basis of the gas-phase radical mechanism. During combustion, they pass into the gas phase to form phosphorus radicals (PO_2_, PO, etc.), which can capture radicals from the combustion of the polymer and thus reduce or even stop the combustion reaction. In addition, phosphorus-based flame retardants can catalyze polymer carbonization [16]. The influence of the chemical structure on the mechanism of action of flame retardants is important not only for small molecules, but also for organophosphate polymers. Aromatic polyphosphonates show higher thermal stability than aliphatic polyphosphonates, and at the same time have higher hydrolytic stability than aromatic polyphosphates. This fact is explained by the presence of a stable bond between phosphorus and carbon P-C in phosphonates, while phosphates have an additional hydrolyzable P-O-C bond, which results in lower degradation temperatures [20].

In summary, there are few publications in the literature on the modification of flame resistance properties of ABS intended for applications in 3D printing technology. In this work, polymer composites based on acrylonitrile-butadiene-styrene with the addition of selected flame retardants were developed and obtained. The introduced additives were selected in such a way as to investigate the effect of the phosphorus flame retardant on the flame resistance of ABS composites and the effectiveness of their use.

## 2. Materials and Methods

### 2.1. Materials

Commercial granulate (Terluran HI-10 natural from Ineos Styrolution Group, Frankfurt am Main, Germany) designated as ABS was used as the polymer matrix. Tetrakis (2,6-dimethylphenyl)-m-phenylene biphosphate (flame retardant) (“PX200” from WTH Walter Thieme Handel GmbH, Stade, Germany) was used as the flame retardant of the material.

The compositions of the individual compositions are summarized in Table 1.

### 2.2. Preparation of the Composites and Sample

The PX200 flame retardant, which was a white powder, was crushed using a ball mill, and then the pre-crushed powder was sieved on laboratory sieves with a mesh size of 0.50 mm and 0.25 mm. The aim of these activities was to obtain a fraction of the flame retardant with a smaller grain diameter, to improve its extrusion and homogenization with ABS granulate at a later stage, and to obtain a better quality filament with the same properties along its entire length.

The ABS granulate was dried (time: 4 h; temperature 80 °C) in a vacuum dryer to remove moisture that could hinder the extrusion process.

Then, appropriate amounts of unmodified ABS granulate and PX200 flame retardant were mixed. The homogenization of the components was carried out using a Coperion ZSK 18 ML twin-screw extruder equipped with a granulation line. Process parameters: screw speed 400 rpm, extrusion capacity 4 kg/h, temperature range 230–245 °C.

The obtained granules were dried in a vacuum oven at 80 °C for 3 h. The thus obtained polymer composites were used to obtain fibers with a diameter of approx. 1.75 ± 0.05 mm on the designed filament production line (Gamart SA, Jasło, Poland) in the temperature range 225–240 °C. These values allowed obtaining a filament with a diameter of 1.7 mm, which is suitable for 3D printing. The diameter of the filament was measured by a measuring device located in front of the spool on which the filament was wound. The designed proprietary technological line is presented elsewhere [21,22].

**Figure 2:**
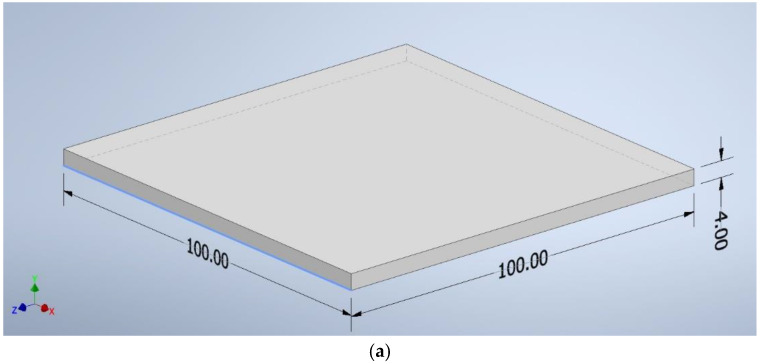

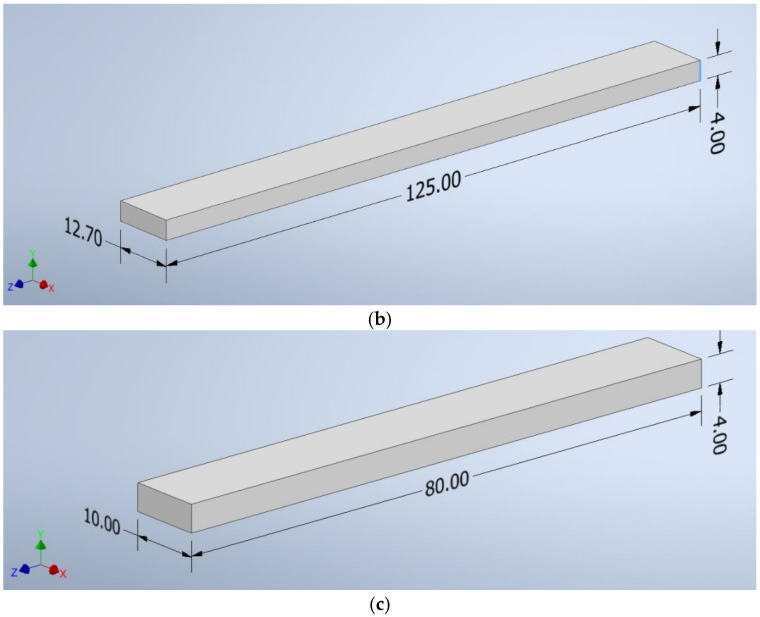
The dimensions of the samples: (**a**) a square of 100 mm × 100 mm × 4 mm, (**b**) a beam of 125 mm × 12.7 mm × 4 mm, (**c**) a beam of 10 mm × 80 mm × 4 mm.

The CAD program created shapes with the following dimensions: a square of 100 mm × 100 mm × 4 mm (Figure 2a), a beam of 125 mm × 12.7 mm × 4 mm (Figure 2b), and a beam of 10 mm × 80 mm × 4 mm (Figure 2c). The file was saved as an “.stl” extension—a ready format for reading by the software controlling the 3D printer.

**Figure 3:**
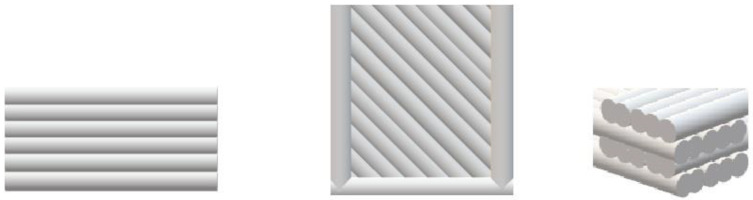
Arrangement of layers, respectively view: front and side of the sample, top and bottom, cross-section of layers.

The composites were used to obtain samples needed for further tests on the 3D printer (UP BOX, TierTime, Beijing, China) in the FDM technology (Figure 3).

The process parameters are summarized in Table 2.

### 2.3. Methods Characterization

The mass flow rate test was performed using a plastometer (DYNISCO 4781, Kayeness INC., Honey Brook, PA, USA). The test consisted of introducing a sample of approximately 4 g into the apparatus heated to the processing temperature (220 °C), the measurement was performed with a load of 10.0 kg. Three measurements were performed for each composite in accordance with the guidelines of ISO 1133 [23].

The studies of the appropriate dispersion of PX200 in the ABS matrix were carried out using a scanning electron microscope, SEM with an Energy Dispersive Spectroscopy, EDS analyzer (Gemini SEM 560 ZEISS, Oberkochen, Germany) with an accelerating voltage of 15 kV and secondary electron detection (SE2). The samples intended for the studies were prepared by making surface microscopes.

The chemical structures of ABS and ABS composites after filament fabrication were characterized using a Alpha FTIR spectrometer (Bruker, Billerica, MA, USA) with an ATR attachment (attenuated total reflection). Three samples from each part were scanned 64 times each in the wavenumber range 4000–400 cm^−1^. The analysis of the spectra obtained was carried out in the Opus software 6.5 (Bruker, Billerica, MA, USA).

The thermal decomposition behaviors of the ABS composites was performed in a TGA/DSC thermogravimeter (METTLER Toledo, Greifensee, Switzerland) with the temperature ranging between 25 °C and 700 °C at a heating rate of 10 °C/min under a nitrogen atmosphere.

**Figure 4:**
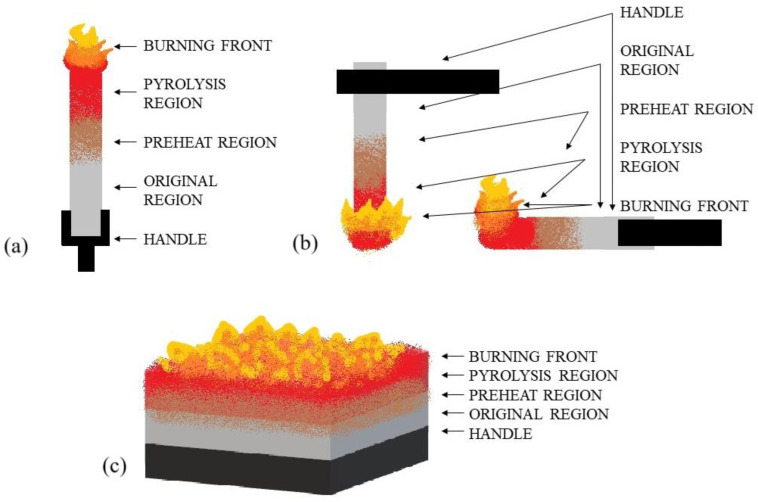
Test scheme: (**a**) LOI, (**b**) UL-94 vertical test and horizontal test, (**c**) mass loss cone calorimeter.

The limiting oxygen index (LOI) for the composites was determined in accordance with EN ISO 4589 [24] at room temperature using a Fire Testing Technology Ltd. (East Grinstead, UK) device. Printed samples of 10 mm × 80 mm × 4 mm were tested. The study scheme is shown in Figure 4a.

The UL94 flammability tests were conducted in a chamber manufactured by FTT Ltd. (East Grinstead, UK). The measurements were performed in accordance with PN EN 60695-11-10 [25] with the sample beam positioned vertically and horizontally and a methane-fuelled burner with a height of 25 mm. Printed samples of 12.7 mm × 125 mm × 4 mm were tested. The study scheme is shown in Figure 4b.

The heat release rate (HRR in kW/m^2^) during combustion of the sample, as well as other parameters characterizing flammability, were evaluated using a mass loss cone calorimeter, a product of FTT Ltd. (East Grinstead, UK), in accordance with ISO 13927:2023 [26], using a heat flux of 50 kW/m^2^ and a distance from the ignition source of 25 mm. Printed samples of a shape of 100 mm × 100 mm × 4 mm were tested. The study scheme is shown in Figure 4c.

The chemical composition of the residue after microcone calorimeter test was characterized with a Alpha FTIR spectrometer (Bruker) for a range of infrared wavelength number values of 4000–400 cm^−1^, using a tablet of powdered residue in KBr.

The chemical composition of the products formed after the decomposition of the composites was analyzed using a TGA Nicolet iZ10 thermal analyzer. The determinations were carried out in a nitrogen atmosphere in the temperature range of 0–700 °C.

## 3. Results and Discussion

### 3.1. Rheological Properties

The viscosity of the melted polymer is a key factor in determining the fire resistance of the material, which has been confirmed by many scientists in their work so far [27,28]. The phenomenon is caused by the ease and length of the melt dripping, because they can reduce the spread of flame or even lead to its extinction, because they remove mass and heat from the actual pyrolysis zone, thus achieving higher results, e.g., LOI. In the literature, data can be found on the obtained MFI results of materials containing flame retardants in their composition. A decrease in the fluidity of the material is observed after the introduction of typical flame retardants (e.g., ammonium polyphosphate (APP), pentaerythritol (PER)), because they act as reinforcing fillers [27,28,29,30]. No data was found in the source texts regarding the tetrakis(2,6-dimethylphenyl)-m-phenylene biphosphate (PX200) used in the work. Table 3 summarizes the average results of the melt flow mass index of the ABS-based composites.

The results presented in Table 3 show that the MRF increased with the increase in the amount of flame retardant in the ABS material. In practice, this meant a higher fluidity of the material. The material had an MFR of 5.94 g/10 min. The addition of 5% and 10% increased the MFR value by 45.62% and 148.99%, respectively. A very large increase in MFR occurred in the case of the addition of 15% and 20% PX200—38.05 g/10 min and 20% PX200—82.59 g/10 min. The highest fluidity, by about 1290%, was characteristic of ABS with the highest tested flame retardant content (20%). The results were satisfactory, because, as already shown in the previous parts of the work, the material that flows more easily promotes the dripping effect, which reduces the spread of flame by moving the melting material away from the flame zone. Moreover, such a significant increase in MFI did not negatively affect the technological processes necessary to be carried out in this work, such as obtaining a filament with a specific, constant shape/diameter.

### 3.2. Structural Properties

In order to confirm the presence and distribution of the inorganic flame retardant PX200 in ABS composites, scanning electron microscopy (SEM) analysis combined with local analysis of the chemical composition using the EDS (Energy Dispersive Spectroscopy) method was performed. The results are summarized in Table 4.

SEM-EDS analysis performed for ABS composite samples filled with PX200 showed the presence of characteristic inorganic elements, such as P (phosphorus) and K (potassium), absent in unmodified ABS. With the increase of PX200 content, a systematic increase in the signal intensity for P and K elements is observed, which confirms the effective introduction of the flame retardant into the polymer matrix. The decrease in the C content and the increase in the O content also confirm the presence of the inorganic phase. Elemental mapping showed a relatively uniform distribution of PX200 particles in the ABS matrix, without any significant aggregation.

### 3.3. Chemical Structure

**Figure 5:**
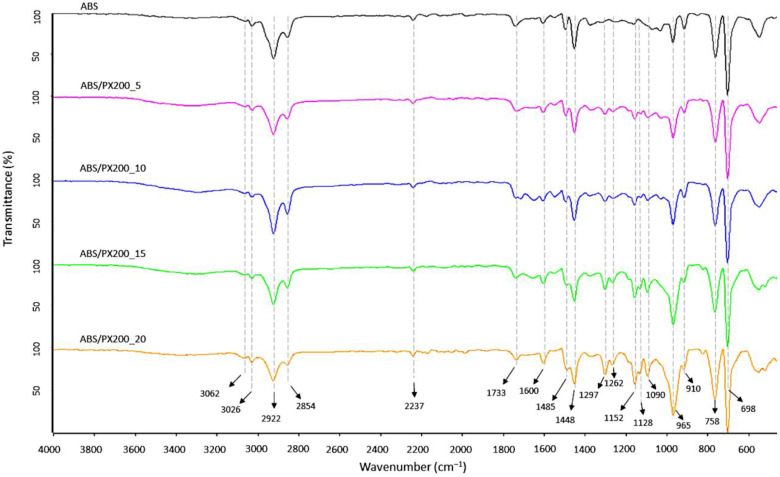
FT-IR spectra of filaments surface fabricated from ABS and ABS containing PX200.

FT-IR analysis of the surface of the produced ABS filaments and ABS with different contents of the flame retardant PX200 was performed, obtaining spectra summarized in Figure 5. According to the literature data, the ABS spectrum shows characteristic absorption bands typical for this terpolymer, such as those related to the stretching vibrations of the C-H bond in the aromatic ring at the maximum wavelength of 3062 and 3026 cm^−1^ and to out-of-plane bending vibrations at the bands of 758 and 698 cm^−1^. In turn, the bands with the maximum of 2922 and 2854 cm^−1^ are related to the stretching vibrations of the antisymmetric C-H bond (aliphatic). Absorption bands at the wavelength of 2237 cm^−1^ related to the CN group in acrylonitrile were also observed. The absorption bands at wavelengths of 1600, 1495 cm^−1^ were assigned to the skeletal vibrations of the aromatic ring in styrene. The presence of a band characteristic of polybutadiene, related to the vibrations of the C=C bond (1448), was also revealed, as well as the bands of 965 and 910 cm^−1^ related to the vibrations of the C-H bond in 1,4-butadiene and 1,2-butadiene, respectively [31,32,33]. In the case of ABS composites with the content of organophosphorus flame retardant, additional absorption bands of radiation were recorded in the range of wave numbers 1297, 1262 and 1152 cm^−1^ related to the P=O group, as well as in the range of wave numbers 1128, 1090 cm^−1^ related to the vibrations of the P-O and P-O-C_Ar_ bonds. In all analyzed samples, there were also observed bands derived from bond vibrations of C=O group (1733 cm^−1^), which according to literature data is related to possible thermomechanical degradation and oxidation of the polybutadiene phase caused by additional extrusion mixing process, forming filament and 3D printing [34] or the presence of sterically hindered phenolic antioxidants [35]. What is important, the intensity of this peak is the same for all samples.

### 3.4. Fire Behavior: Forced Flaming Combustion

**Figure 6:**
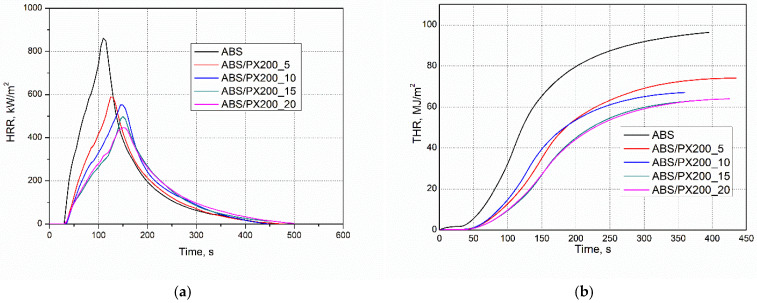
Representative curves: (**a**) HRR and (**b**) THR vs. time for ABS and ABS-based composites.

The combustion behavior and potential fire hazard of ABS composites were conducted using micro cone calorimeter. The results for the individual combustion indices were generated using the “MLC Calc” program, both in the form of numerical data and graphs. The results are presented in Table 5 and Figure 6a,b.

It was found that the reduction of HRR, and in particular the peak HRR measured by cone calorimetry, is the clearest evidence of the effectiveness of the flame retardant [36,37,38,39,40]. Unmodified ABS is characterized by the highest value of the average heat release rate, amounting to 268.91 kW/m^2^. Materials with a high HRR value, i.e., in this case the polymer matrix not modified with a flame retardant, release heat more intensively during combustion (Figure 6a). This condition can catalyze further cleavage of bonds between polymer chains, leading to faster decomposition, which facilitates the spread of fire [36,37]. The addition of phosphorus flame retardant to the polymer matrix reduced the HRR value for each of the obtained composites, even by about 113 units for ABS/PX200_5. Moreover, the addition of the flame retardant has a positive effect on extending the time that elapses before the moment of maximum heat release rate (T pHRR) occurs. It was observed that the higher the amount of flame retardant, the higher the T pHRR value. ABS has the lowest T pHRR value of 115 s, while the best ABS/PX200_20 composite had a time of 157.5 s.

### 3.5. Fire Behavior: Quantitative Assessment of Flame-Retardancy Mechanisms

Using cone microcalorimeter data, the effect of reducing THR and pHRR was quantified for obtained ABS composites according to calculations based on our work and literature data [41,42]. The activity of the introduced flame retardants in the gas phase was determined by determining the effective heat of combustion (EHC). In turn, the ability of the composites to carbonize in the condensed phase is determined by the mass loss after combustion (PML). A higher charring yield reduces the rate of release of flammables and volatiles. Also, a reduction in the effective heat of combustion of EHC indicates flame inhibition or fuel dilution in the combustion zone [43]. For the ABS/PX200_5 composite, the EHC value was reduced to 78.3% compared to the ABS sample. I turn, the residue decreased from 5.1% to 0.5% 0.5% instead of 5.1%) In contrast, the combustion residue decreased from 5.05% to 0.55%, corresponding to an increase in flammable gas release up to 104.7%. Based on the THR value, which was reduced to 85.5%, the gas-phase activity plays a major role in its reduction as confirmed by the calculated value (78.3% × 104.7% = 82.1%). In turn, the pHRR was reduced to 70.5%. According to calculations (78.3% × 104.7% × 85.9 = 70.5%), this is due to an additional barrier effect (14.1%). Probably, it is due to the formation of carbonized layer in the initial phases of combustion, which is thermally unstable and has decomposed, since no residue was observed in the cone microcalorimeter after combustion. This is also indicated by the continuously increasing value of THR as a function of time (Figure 6b). Similar relationships were obtained for composites with higher content of PX200 (Figure 5). Regardless of the flame retardant content, the residue after combustion was smaller than for ABS, and the reduction in THR is due to its effect in the gas phase (32%). Once again, additional reduction in pHRR to 63.6, 63.5 and 59.7% for ABS/PX200_10, ABS/PX200_15 and ABS/PX200_20, respectively, can be caused by the barrier effect in the initial stage of combustion, as calculated (9.8, 10.3 and 16.1%, respectively).

### 3.6. Fire Behavior: Characteristics of Fire Spread and Efficiency of Flame Retardants

The THR index value ambiguously determines the composite’s resistance to fire. In addition, the fire process conditions determined in the cone microcalorimetry test constitute a limitation in the correct assessment of the fire characteristics and flame spread. Also, the value of the average heat release rate HRR as a function of fire time, in which the pyrolysis front moves in the thickness of the ABS composite sample, does not characterize the actual way the flame spreads over its surface. Therefore, in order to correctly assess the functional properties of polymers that reduce their flammability during a fire, several phenomena occurring during a fire were characterized, which determine its different course. In order to quantitatively and qualitatively analyze the fire spread and the effectiveness of the phosphorus flame retardant PX200 in the produced composites, the following indices were adopted:

- MARHE [kW/m^2^]—Maximum average rate of heat emission—characterizes the potential spread of fire or flame. This value was read from ARHE (Average Rate of Heat Emission) graphs generated by the “MLC Calc” program.
- FIGRA [kW/m^2^·s]—Fire Growth Rate Index—determines the ability for fire to occur. It is defined as the maximum value of HRR over the time of occurrence of this value, as shown in the following formula:

(1) $$FIGRA=\frac{pHRR}{T\;pHRR}[kW/m^{2}\cdot s]$$

- FPI [m^2^·s/kW]—Fire Potential Index—assessment of the efficiency in forming a scale of the tested composites. Defined as the time to ignition over the maximum HRR value:

(2) $$FPI=\frac{TTI}{pHRR}[m^{2}\cdot s/kW]$$

- FRI [-]—Flame Retardancy Index—quantitative assessment of the effectiveness of the flame retardants used. Defined as the relationship:

(3) $$FRI=\frac{[THR\cdot \frac{pHRR}{TTI}{]\;}_{polymer}}{[THR\cdot \frac{pHRR}{TTI}{]}_{polymer+flameretardant}}\;$$

The obtained results of the calculations of the indicators are presented in Table 5.

Analyzing the data (Table 6) concerning the MARHE index, it was found that the addition of a flame retardant reduces the maximum average rate of heat emission. The lowest value was achieved for ABS shapes modified with the addition of 15% PX200 and it is 232.0 kW/m^2^. The highest MARHE value was shown by ABS with the result of 395.0 kW/m^2^. There is no correlation between the amount of flame retardant and the MARHE value. Based on these results, it was found that materials modified with the addition of a phosphorus flame retardant are characterized by a lower potential ability to spread fire or flame. The FIGRA fire growth rate index decreased more than twice after modifying the ABS material with the addition of 20% PX200. The FIGRA value for this composite is 3.057 kW/m^2^*s compared to 7.069 kW/m2·s for ABS. The addition of a flame retardant therefore reduces the ability to cause fire even more than twice. The fire potential index FPI increased its value after the flame retardant was used. The highest value was achieved for the 20% PX200 additive and it is 0.061 m^2^·s/kW compared to 0.037 m^2^·s/kW for ABS. It can therefore be stated that the flame retardant additive increases the efficiency of coal scale formation, which makes the composite combustion more difficult. The fire retardancy index FRI increased after the addition of the phosphorus flame retardant. In the case of 5%, 15% and 20% PX200 additives, the increase compared to ABS is more than twofold. The highest value was achieved for the 15% PX200 additive and for this composite the FRI value is 2.128. The composite filled with 5% PX200 achieved a smaller increase—its FRI value is 1.467. The calculated FRI values are the basis for classifying the efficiency of the phosphorus flame retardant used on a three-point scale: poor (FRI < 1), good (FRI = 1–10) and excellent (FRI > 10). Based on this scale, it was found that the phosphorus flame retardant used demonstrates good efficiency.

### 3.7. Flammability

In order to determine the response to a direct fire source and to demonstrate the fire suppression capability of ABS composites, limiting oxygen index LOI and UL94 chamber tests were conducted.

**Figure 7:**
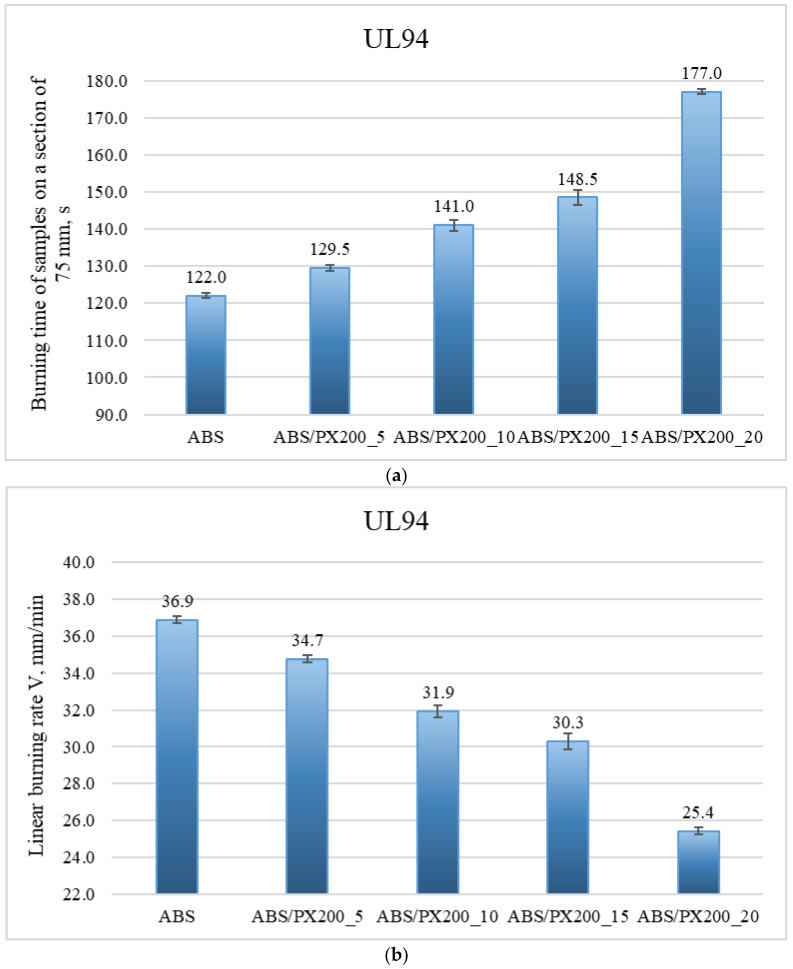
Results of the horizontal UL94 test, which determined the burning time of samples in the form of beams over a distance of 75 mm (**a**) and the linear burning rate (**b**).

The UL-94 test was performed in two sample positions vertically (all materials were completely burned—negative test) and horizontally. The graphs below (Figure 7a,b.) show the results of the horizontal UL94 test, in which the burning time of samples in the form of beams over a distance of 75 mm and the linear burning rate were determined.

Based on the graph of the burning time of samples on a 75 mm section, it was found that with the increase in the amount of flame retardant, the burning time of the material in the horizontal UL94 test increases. The best result was again achieved with the addition of 20% PX200—the burning time of the sample with the addition of flame retardant at this content was 177 s. The worst result was achieved by ABS—the burning time of the material was 122 s. These results translate into the linear burning rate, which was calculated based on the following formula:

(4) $$V=\frac{60\cdot L}{t}[mm/min]$$

where:

L—length at which the sample was destroyed [mm]
t—sample burning time [s]

All samples burned over the entire designated section, therefore L = 75 mm. The profile with the addition of 20% PX200 burned the slowest—the result was 25.42 mm/min (the best result). The ABS sample gave a result of 36.89 mm/min. Based on the results of the linear burning rate, individual composites were assigned to the appropriate flammability class in the UL94 horizontal test. The materials were classified as HB40 flammability class—linear burning rate below 40 mm/min. The longer burning time of the profile and the lower burning rate indicate improved fire properties after adding the flame retardant and a lower risk of fire spreading. In addition, a greater effect of melt dripping was observed during the test for composites with 15% and 20% PX200 content. Melt dripping reduces the spread of flame by moving the melting material away from the flame zone. By introducing a flame retardant into the polymer matrix, the breaking of polymer chains is accelerated, which facilitates the melting of the burning polymer. By introducing a flame retardant, the rheology of the material is affected, reducing its viscosity, which was also demonstrated in the MFR test described earlier in the paper. The higher the flame retardant content in the composite, the lower its viscosity, which caused a greater dripping effect.

In order to evaluate the effect of PX200 addition on the flammability properties of ABS material, a one-way analysis of variance (ANOVA) was performed based on the UL94 test results. ABS and composites containing 5%, 10%, 15% and 20% of PX200 addition were compared. The results of the analysis showed that the differences between the groups were statistically significant: F-statistic = 569.94; *p*-value = 0.00000078 (*p* < 0.05). This means that the PX200 content has a significant effect on the burning time of the samples in the UL94 test. A clear trend is observed: with the increase in the amount of PX200, the burning time of the material is significantly extended, which may indicate an improvement in fire resistance.

**Figure 8:**
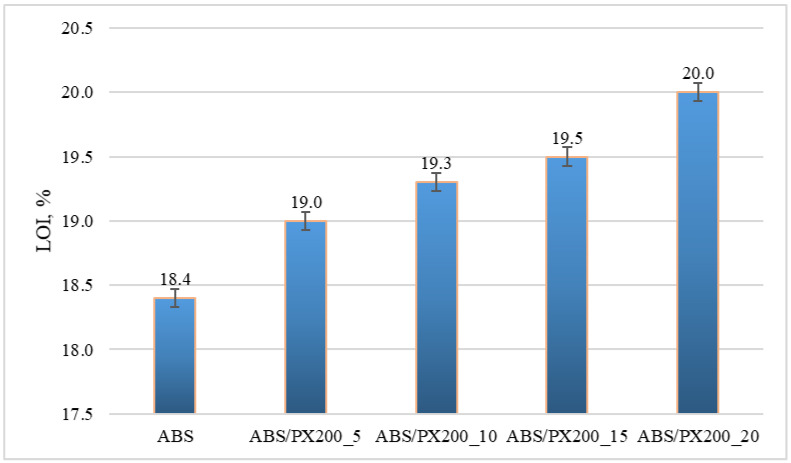
Limiting oxygen index LOI for ABS and ABS containing flame retardant.

The LOI value represents the minimum concentration of oxygen in the oxygen and nitrogen mixture that will sustain the flaming combustion of the material. The unmodified polymer matrix shows very poor flame retardant properties, similar results were also obtained by other researchers [37,38]. It was observed that a larger amount of PX200 in the composite caused an increase in the oxygen concentration in the oxygen and nitrogen mixture needed to sustain the combustion of this composite. The material with the addition of 20% PX200 showed an LOI of 20% (Figure 8). The lowest index of 18.4% was obtained for the ABS matrix. Materials for which the LOI value is below 21% are considered self-igniting, while those with the LOI value above 21% are self-extinguishing in atmospheric conditions. Therefore, all composites obtained in this work can still be considered self-igniting, which indicates that the phosphorus flame retardant used had too weak an effect on the obtained LOI results.

Also in this case, the results of the one-way analysis of variance (ANOVA) showed that the differences between the groups are statistically significant: F = 141.2; *p* < 0.00003. This means that the PX200 content has a statistically significant effect on the LOI value. With the increase in the PX200 content, a systematic increase in the LOI value is observed, which suggests an improvement in the fire resistance of the material.

### 3.8. Analysis of Flame Retardant Mechanism

#### 3.8.1. Thermogravimetric Analysis

**Figure 9:**
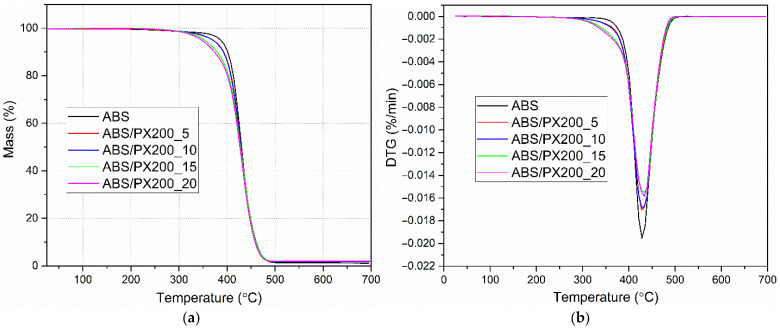
Mass change curves (**a**) and mass change derivative curves (**b**) of ABS and ABS-based composites.

Based on the thermogravimetric analysis conducted in a nitrogen atmosphere, the effect of different contents of the organophosphorus flame retardant PX200 on the thermal stability of ABS was determined. The obtained curves in the form of changes in the sample mass as a function of temperature TG allowed to determine the temperature of 5% mass loss, which determines the thermal stability of the composites and the residues after degradation m_700_. In turn, the DTG curves were used to determine the temperature for the maximum degradation rate (T_max_) and mass change during the stage of maximum degradation rate ∆m. The obtained results are presented in Table 7 and Figure 9a,b. The analysis of the results indicates a single-stage ABS decomposition process, which is consistent with the literature data [44,45]. The addition of the flame retardant does not change the nature of the decomposition. On the other hand, a significant decrease in thermal stability of ABS/PX200 composites was observed, as evidenced by a decrease in the temperature at which 5% mass loss occurs. With the increase in the flame retardant content, the value of this parameter changes from 386.4 °C for ABS to 346.0 °C for ABS/PX200_20. A similar significant decrease in the thermal stability of polymers containing organophosphorus flame retardants was observed by the authors of other papers [46]. In turn, the temperature at which the maximum rate of mass loss occurs changes slightly towards higher values—an increase of 4.6 °C for the composite with 20% PX200 content. As a result, no scale is formed after pyrolysis at 700 °C, which indicates complete evaporation of the flame retardant into the gas zone. The same conclusions were presented in a paper by Sanchez and Villanueva, who tested organophosphorus flame retardants in ABS/PC blends [47]. The obtained results are consistent with the combustion data in a cone microcalorimeter, where the mass loss was at the level of 99%.

#### 3.8.2. Char Residue Analysis

**Figure 10:**
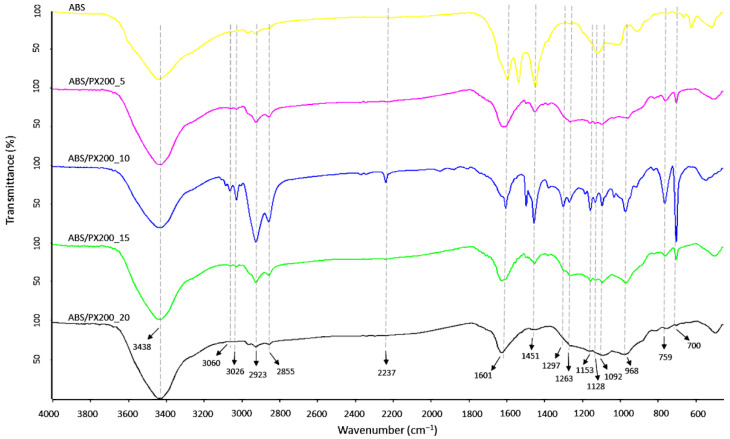
FT-IR spectra of char residue after mass loss calorimeter test of ABS and ABS composites.

In order to verify the results obtained during TG analysis and combustion in a cone microcalorimeter, a qualitative analysis of the scale collected after combustion of the composites in the MLC was carried out. A new broad band with a maximum of 3438 cm^−1^, assigned to the O-H group stretching vibrations, appeared in the spectrum of all samples (Figure 10), which is consistent with the literature data [48,49]. Moreover, in comparison to the ABS spectrum before combustion, there was a decrease in the intensity of the bands characteristic for ABS such as: 3060, 3026 cm^−1^ (C-H aromatic ring), 2923 and 2855 cm^−1^ (C-H aliphatic), 759 and 698 cm^−1^ (CH_2_ aromatic ring in styrene). Moreover, a complete disappearance of the 2237 cm^−1^ band from the CN stretching vibrations was observed, as well as the 910 cm^−1^ band associated with the C-H bond vibrations in 1,2-butadiene, which indicates significant pyrolysis of the butadiene part. In the case of the FT-IR spectrum of the residue formed after combustion of ABS with flame retardants, the intensity of absorption bands also decreased in comparison to the composites before combustion. Also the peaks indicating the presence of P=O groups (1298, 1263, 1153 cm^−1^), P-O and P-O-Ar (1128 and 1092 cm^−1^) decreased. Analysis of the post-combustion residue spectra confirmed the lack of activity of the PX200 flame retardant in the condensed phase—no peaks of polyaromatic and cross-linked P-O-P structures (1000, 920 cm^−1^) [50].

## 4. Conclusions

The aim of the work was to develop polymer composites based on ABS with increased flame resistance, which are suitable for applications in 3D printing. For this purpose, appropriate additives were selected—flame retardants, which was tetrakis (2,6-dimethylphenyl)-m-phenylene bisphosphate with the trade name PX200. The flame retardant additive was dispersed in the polymer matrix in various amounts (5 wt.%, 10 wt.%, 15 wt.% and 20 wt.%). The effect of the presence and amount of the added additive on the rheological properties (mass melt flow rate) and flame resistance (cone microcalorimeter, UL-94 test, limited oxygen index) of ABS was determined. Composites with PX200 were characterized by significantly increased fluidity compared to ABS (5.94 g/10 min). With the increase in the content of the additive in the polymer matrix, the MFR of the composites increased, the highest result, by as much as 1290% higher compared to ABS, was obtained for the ABS/PX200_20 composite (82.59 g/10 min). Such high fluidity of materials is often the cause of failures in many processing methods, in this case the high MFR of the composites did not cause technological problems at the stages of work, primarily at the stage of obtaining the filament necessary to print samples in the material extrusion technology. The SEM-EDS results confirmed the effective introduction of the flame retardant into the polymer matrix. The addition of the phosphorus flame retardant PX200 brought about a moderate improvement in the indicators of its effectiveness and determining the potential course of fire. The shapes modified with 15% PX200 are characterized by the lowest potential ability to spread fire or flame (MARHE index equal to 232.0 kW/m^2^). The fire risk decreased by more than twofold after modifying the ABS material with the addition of 20% PX200—the value of the FIGRA fire growth rate index for this composite is 3.057 kW/m^2^·s. The addition of PX200 increases the efficiency of coal scale formation, which makes the combustion of the composite more difficult. This is confirmed by the FPI fire potential index. For the addition of 20% PX200, it is the highest (0.061 m^2^·s/kW). The addition of PX200 delays the combustion of the composite by more than twofold—the FRI combustion delay index for the ABS/PX200_15 composite is 2.128, which corresponds to the good efficiency of the flame retardant used on a three-point scale. The composite containing the addition of 15% phosphorus flame retardant shows the greatest inhibition of combustion in the gas phase. For the composites obtained, there is no charring in the condensed phase—PX200 only works in the gas phase, which means that the improvement in flame resistance is small. The formation of a protective scale layer is the greatest for the composite with the addition of 20% flame retardant and amounts to 24.447%. With the increase in the amount of PX200 in the composite, the burning time of the shapes in the horizontal UL94 test increases. The best result was shown by the addition of 20% PX200—the burning time of the sample with flame retardant with this content was 177 s. All materials were assigned the HB40 flammability class—linear burning rate below 40 mm/min. The longer burning time of the shape and the lower burning rate indicate an improvement in fire properties after adding the flame retardant and a lower risk of fire spreading. The limiting oxygen index LOI value increased from 18.4% for unmodified ABS to 20% for materials with 20% of PX200 flame retardant. Unfortunately, all composites obtained in this work were still classified as spontaneously combustible (LOI < 21%), which indicates too weak effect of the phosphorus flame retardant used. The influence of different contents of organophosphate flame retardant PX200 on thermal stability of ABS was also determined. Analysis of mass change curves and mass change derivative curves indicates a single-stage decomposition process of ABS, the addition of flame retardant did not change the nature of decomposition. Moreover, a decrease in thermal stability of ABS/PX200 composites was observed, with the increase of flame retardant content the value of this parameter changes from 386.4 °C for ABS to 346.0 °C for ABS/PX200_20. Despite this, the temperature at which the maximum rate of mass loss occurs changes slightly towards higher values (an increase of 4.6 °C for ABS/PX200_20). As a result, no residue was formed after pyrolysis at 700 °C, which indicates complete evaporation of the flame retardant to the gas phase. FT-IR analysis of the char residue collected after combustion of the composites in MLC confirmed the lack of activity of the PX200 flame retardant in the condensed phase.

## Figures and Tables

**Table 1:** ABS compositions with flame retardants.

Sample Symbol	ABS Content (wt.%)	PX200 Content (wt.%)
ABS	100	-
ABS/PX200_5	95	5
ABS/PX200_10	90	10
ABS/PX200_15	85	15
ABS/PX200_20	80	20

**Table 2:** Compositional data of the composites.

Printing Parameters	
Nozzle diameter, mm	0.4
Layer height, mm	0.2
Infill percentage, %	100
Infill pattern, °	±45
Extrusion temperature, °C	275
Bed temperature, °C	105
Printing speeds, mm/s	45

**Table 3:** Summary of the obtained MFR results.

Sample Symbol	ABS	ABS/PX200_5	ABS/PX200_10	ABS/PX200_15	ABS/PX200_20
MFR [g/10 min]	5.94 ± 0.12	8.65 ± 0.25	14.79 ± 0.08	38.05 ± 1.84	82.59 ± 2.05

±standard deviation.

**Table 4:** Distribution of selected elements K, P, O and C in the structure of 3D printed ABS-based composite samples.

Sample Symbol	Distribution of the Elements K, P, O and C
**ABS**	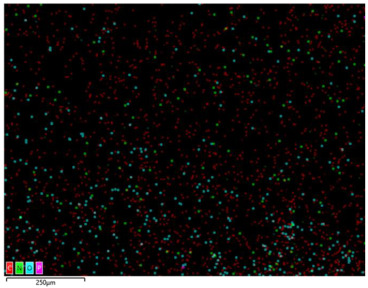
**ABS/PX200_5**	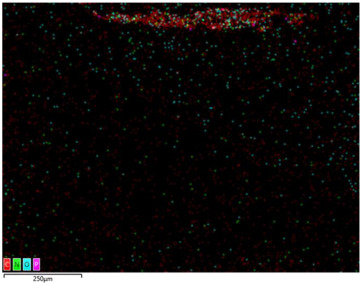
**ABS/PX200_10**	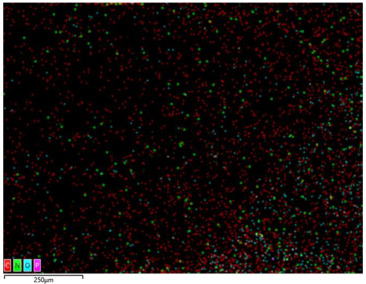
**ABS/PX200_15**	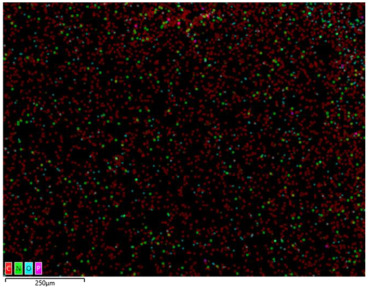
**ABS/PX200_20**	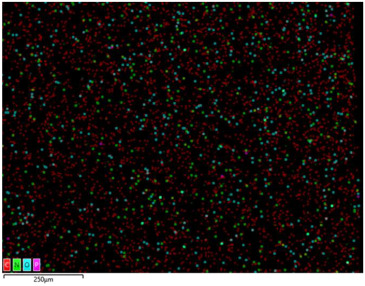

**Table 5:** Summary of average results of the main indices obtained from the cone microcalorimeter test.

Sample Symbol	HRR [kW/m^2^]	pHRR [kW/m^2^]	T pHRR [s]	THR [MJ/m^2^]	TTI [s]	EHC [MJ/kg]	PML [%]
**ABS**	268.9 ± 4.5	808.7 ± 83.8	115.0 ± 7.1	86.5 ± 10.8	30.0 ± 2.8	22.3 ± 3.0	94.9 ± 2.2
**ABS/PX200_5**	155.9 ± 33.9	569.8 ± 24.6	140.0 ± 7.1	74.0 ± 0.3	27.0 ± 5.6	17.5 ± 0.1	99.5 ± 0.4
**ABS/PX200_10**	183.6 ± 39.0	514.2 ± 84.0	132.5 ± 10.6	62.4 ± 5.	28.0 ± 5.6	15.2 ± 2.3	98.5 ± 0.2
**ABS/PX200_15**	185.8 ± 22.4	513.2 ± 39.1	152.5 ± 3.5	64.7 ± 5.9	30.0 ± 0.0	15.1 ± 0.9	99.3 ± 0.2
**ABS/PX200_20**	174.7 ± 21.5	482.9 ± 73.2	157.5 ± 10.6	68.4 ± 6.5	29.0 ± 0.0	15.2 ± 1.3	99.3 ± 0.4

±standard deviation. where: HRR—Heat Release Rate; pHRR—peak Heat Release Rate; T pHRR—Time of peak Heat Release Rate; THR—Total Heat Released; TTI—Time to Ignition; EHC—Effective Heat of Combustion; PML—Percentage Mass Loss.

**Table 6:** Summary of average results of calculated indicators.

Sample Symbol	MARHE [kW/m^2^]	FIGRA [kW/m^2^·s]	pHRR/TTI [kW/m^2^·s]	FPI [m^2^·s/kW]	FRI [-]
**ABS**	395.00 ± 62.23	7.069 ± 1.16	27.21 ± 5.36	0.037 ± 0.010	1.000 ± 0.330
**ABS/PX200_5**	279.50 ± 12.02	4.080 ± 0.38	21.68 ± 5.45	0.048 ± 0.010	1.467 ± 0.390
**ABS/PX200_10**	253.00 ± 29.70	3.919 ± 0.95	18.44 ± 0.72	0.054 ± 0.000	2.045 ± 0.100
**ABS/PX200_15**	232.00 ± 8.49	3.363 ± 0.18	17.11 ± 1.30	0.059 ± 0.000	2.128 ± 0.360
**ABS/PX200_20**	234.00 ± 16.97	3.057 ± 0.26	16.65 ± 2.53	0.061 ± 0.010	2.068 ± 0.520

±standard deviation. where: MARHE—Maximum Average Rate of Heat Emission; FIGRA—Fire Growth Index pHRR/TTI—indicator for developing a Petrella chart; FPI—Fire Performance Index; FRI—Flame Retardancy Index.

**Table 7:** TGA data of ABS and ABS composites.

Sample Symbol	T_5%_, °C	T_max1_, °C	Δm_1_, %	m_700_, %
**ABS**	386.4	428.2	98.5	0.0
**ABS/PX200_5**	370.2	428.8	97.9	0.0
**ABS/PX200_10**	369.9	428.8	97.9	0.0
**ABS/PX200_15**	351.6	433.8	97.7	0.0
**ABS/PX200_20**	346.0	432.9	97.7	0.0

## Data Availability

The original contributions presented in this study are included in the article. Further inquiries can be directed to the corresponding author.
